# Management of juxta articular giant cell tumors around the knee by custom mega prosthetic arthroplasty

**DOI:** 10.4103/0019-5413.32045

**Published:** 2007

**Authors:** Mayil Vahanan Natarajan, R Prabhakar, Sameer M Mohamed, RA Shashidhar

**Affiliations:** Department of Orthopedic Surgery, Madras Medical College and Govt. General Hospital, Chennai - 600 003, India; * M. N. Orthopedic Hospital, Chennai, India

**Keywords:** Custom mega prosthesis, juxta articular giant cell tumour, outcome

## Abstract

**Background:**

Juxtaarticular giant cell tumors around the knee are common and pose a special problem of reconstruction after tumor excision. This article analyzes the functional outcome after resection of juxtaarticular giant cell tumors around the knee and replacement by custom mega prosthetic arthroplasty.

**Materials and Methods:**

One hundred and forty-three patients with juxtaarticular giant cell tumors around the knee with mean age of 30.8 years (range 15 to 64 years) underwent resection and replacement by custom mega prosthetic arthroplasty during the period 1994 to 2005. Eighty-one patients were males and 62 were females. Fourteen patients were in Enneking Stage 2 while 129 patients were in Stage 3. Distal femur was involved in 87 patients and proximal tibia in 56 patients. Forty patients presented with pathological fracture at the time of diagnosis. The technique of sleeve resection of the quadriceps musculature was followed to achieve local clearance in distal femoral tumors, and for proximal tibial lesions resection of the tumor-bearing part and a medial gastronemius rotation flap was used routinely. The prosthesis used was a rotating hinge custom mega prosthesis manufactured locally.

**Results:**

The mean follow-up was 5.4 years (1.5 years to 11 years). Functional results were analyzed using Enneking criteria. Excellent results were obtained in 90 patients (62%) and 39 patients had good (27%) results. Periprosthetic fracture (8.3%) and infection (6.9%) were the most common complications followed by aseptic loosening (4.2%). Recurrence of lesion was found in only one patient (0.69%) who was managed with wide local excision.

**Conclusion:**

Custom mega prosthetic arthroplasty is effective in achieving the desired goals of reconstruction with good functional results and least complications in selected patients.

Giant cell tumors (GCT) of bone are aggressive, potentially malignant lesions. It remains a difficult and challenging management problem because there are no absolute clinical, radiographic or histological parameters that accurately predict the tendency of any single lesion to recur or metastasize.^1^ They represent 3-4% of all primary tumors of bone, occurring in young healthy adults in the third and fourth decade of life. The ends of long bones in skeletally mature individuals are involved in more than 80% of cases and 75% of them occur around the knee joint.^2^ Eighty per cent of the GCT have a benign course, with a local recurrence rate of 10-50%; about 10% of GCT undergo malignant transformation through their recurrences and 1-4% give pulmonary metastases even in case of a benign histology.^3^,^4^

The ideal aim in the management of GCT is to eradicate the tumor and still save the joint.^1^ Wide resection is the treatment of choice, especially for situations such as pathological fractures, recurrences and tumors which are high-grade or frankly malignant tumours.^1^,^5^ En bloc resection of major joints creates a problem for the reconstruction of large bone gaps and requires a facility with reconstruction techniques including the use of allografts, large autogenous grafts and fusion or custom arthroplasty. These are technically difficult procedures with many early and late complications. Progress in biomedical engineering along with better surgical techniques has improved overall 10-year prosthetic survival rate after endoprosthetic replacement from 20% to 80% in the past three decades.^6^–^8^ We present here our experience over a decade with custom mega prosthetic arthroplasty for juxta articular giant cell tumors around the knee.

## MATERIALS AND METHODS

Two hundred and five patients of Juxta articular giant cell tumor around the knee were treated. Sixty-two patients in early stage of disease were treated by curettage with bone grafting / cementation. One hundred and forty-three patients with Juxta articular giant cell tumor around the knee who underwent resection and replacement by custom mega prosthetic arthroplasty during the period 1994 to 2005 were analyzed in this study. Those patients who were treated by curettage and bone grafting/cementation during the same period were excluded. There were 81 males and 62 females (ratio of 1.3:1). The age group of the patients was average of 31 years (15 to 64 years). All the patients were histologically proven either by FNAC or open biopsy. Distal femur was involved in 87 patients and proximal tibia in 56 patients. Among the 143 patients, 40 patients presented with pathological fracture at the time of diagnosis. Surgical staging was done according to Enneking's staging system.^9^ Fourteen patients were found to be in Stage 2 of which nine were recurrent lesions after primary surgery elsewhere and 129 patients were in Stage 3.

### The prosthesis

**Figure 1:**
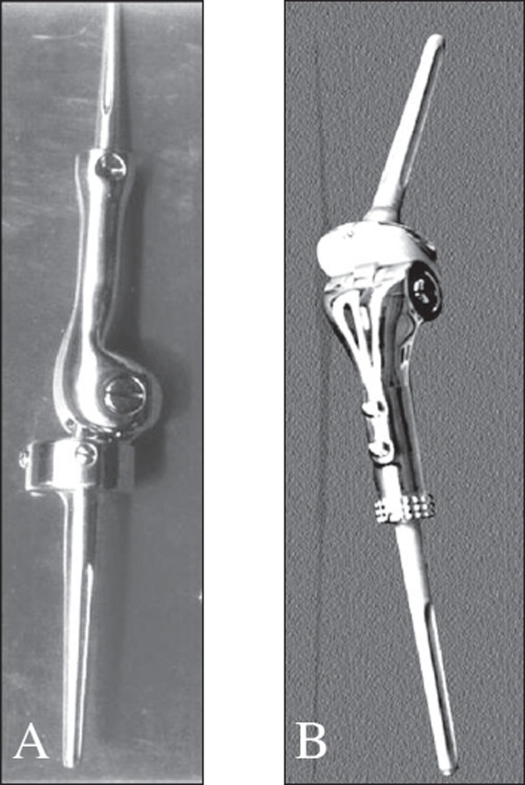
A) Distal femoral prosthesis with rotating axis mechanism, B) Proximal tibial prosthesis with rotating axis mechanism

The rotating hinge custom mega prosthesis, manufactured in Chennai, India was used in all patients. The design has been modified and upgraded over the years. The present design being used is the prosthesis with thrust-bearing pad and rotating axis mechanism. The basic components of the prosthesis are a femoral shaft, a condylar component, a median component, a thrust-bearing pad, a pivot pin, collar bushes and tibial component [Figure 1]. Proximally, the prosthesis is angulated laterally by 6° to resemble the anatomical axis of the lower limb. The function of the thrust-bearing pad is to impart a flexion of 150° between the femoral and tibial components. The ultra-high-molecular-weight polyethylene-bearing pad serves to relocate the load transmitted during weight-bearing. The rotating axis mechanism provides 3° of rotation between the femoral and tibial component. Measurement radiography, CT scans and in some cases MRI were used to estimate the size of the prosthesis to be used. In 119 (83%) patients, 316L stainless steel was the material used to manufacture the prosthesis while titanium alloy was used in the remainder. Expandable custom mega prosthesis was used in one skeletally immature patient.

### Surgical technique

Extended medial parapatellar approach encircling the biopsy scar was used in all our cases. This approach aids in vascular dissection, so that the popliteal vessels can be isolated and the tumor dissection carried out. For distal femoral tumors we used the technique of sleeve resection of quadriceps musculature. The main objective of this technique is to excise a sleeve of quadriceps musculature all around the tumor but retain the functioning rectus femoris tendon. The excision removes a portion of the vastus lateralis, medialis and intermedius, as is deemed necessary in the particular case, but preserves enough musculature to provide soft tissue cover for the prosthesis and retain adequate extension power. By this technique, we were able to attain a balance between achieving an adequate surgical margin and retaining sufficient functioning musculature. For proximal tibial lesions resection of the tumor bearing part and a medial gastrocnemius rotation flap was used routinely in all patients. The extensor mechanism was repaired by direct suturing of the patellar tendon to the transposed flap.

The margin of resection was wide in 133 (93%) and marginal in 10 (7%) patients. The average length of the lesion was 98 mm (70 mm to 200 mm).

## RESULTS

The minimum follow-up was 18 months and the maximum follow-up was 132 months with an average of 65 months. Functional results were analyzed using Enneking criteria.^10^ This system was developed as a clinically based evaluation tool to measure functional outcome in patients with musculoskeletal tumors. It is based on seven primary factors; motion, pain, stability, deformity, strength, functional activity and emotional acceptance, and the criteria for ratings are excellent, good, fair and poor.

Excellent results were obtained in 90 (62%) patients, good in 39 (27%), fair in seven (5.5%) and poor in seven (5.5%). The mean extensor lag for proximal tibial reconstruction was 18° (10°-35°). On analyzing the oncological outcome 142 patients had no evidence of disease and only one patient (0.69%) had recurrence which was managed with wide local excision. There was no case of metastasis or death. The Kaplan Meier estimator^11^ was used to calculate the 10-year survival. Limb survival at 10 years was 97.3% and prosthesis survival was 89.8%.

Both biological and mechanical complications occurred in 31 patients. Infection occurred in nine (6.9%) patients of whom four responded to wound lavage and antibiotics while prosthesis had to be removed in three and amputation was done in two. Flap necrosis occurred in two patients requiring implant removal in one patient and amputation in the other. Periprosthetic fractures occurred in 12 patients (8.3%), of whom six required additional surgical procedures while prosthesis had to be removed in six. Fracture incidence was high in the early part of the series, which declined over the years, probably due to advances in implant design and manufacture. Prosthesis-related failures were observed in two patients requiring revision. Aseptic loosening was present in six (4.2%) patients four of whom underwent revision and two had excellent functional outcome despite evidence of loosening. Overall prosthesis removal was done in 11 patients and amputation was done in three patients.

## DISCUSSION

The problem of selecting the proper treatment in GCT is complicated by the failure of its histological and radiological appearance to indicate its biologic behavior.^12^,^13^ The management of juxta-articular giant cell tumors around the knee occurring in young patients continues to be one of the most challenging areas in orthopedic oncology.^14^ Enneking's and Campanacci's radiographic classifications and surgical staging are helpful in planning the initial surgical treatment, as a number of the active (Stage 2) lesions and most of the aggressive (Stage 3) lesions have a higher incidence of local recurrence (20-50%) when treated by curettage with or without bone grafting.^15^–^17^ The use of methylmethacrylate cement has equivalent recurrence rates.^18^,^19^ The safety and advantages of additional adjuvant treatment of the bone bed with phenol or liquid nitrogen after tumor resection is questionable.^20^,^21^ Since the local behavior of giant cell tumors can be aggressive and they have a greater risk of local recurrence, some authors advocate en bloc resection and reconstruction for these Grade III lesions from the point of view of preventing local recurrence rate and preserving joint.^22^,^23^

Although it is the treatment of choice for these tumors, wide resection creates a problem for the reconstruction of large bone gaps. The reconstructive procedure has to be based on several considerations, such as durability of the surgical procedure, the oncological prognosis, restoration of the anatomy and function, and the needs of the patient.^8^ Rotationplasty gives excellent functional results but the cosmetic outcome is a serious disadvantage of this procedure.^24^ Resection arthrodesis achieves excellent stability but has the major drawback of lack of knee motion.^25^ Massive osteochondral allografts are popular alternative to prosthesis and have been used for benign and low-grade malignant tumors but have several complications like a high rate of infection, fracture of the allograft, nonunion and joint instability.^26^,^27^

The use of mega prosthesis has become the method of choice after bone tumor resection at the knee.^28^ It is the primary modality in the management of malignant bone tumors of lower limb.^29^ Custom mega prosthesis has proved to be a simple, technically superior method of replacing the lost segment of the bone in benign aggressive lesions with pathological fractures and where disease progression has resulted in a clinical situation that prevents skeletal reconstruction after intralesional curettage.^29^,^30^ The advantages of custom mega prosthetic arthroplasty are cost-effectiveness, early resumption of knee function with unassisted ambulation and least rates of recurrence. The possible complications include flap necrosis, secondary infection, aseptic loosening fracture and breakage.^31^,^32^

**Figure 2:**
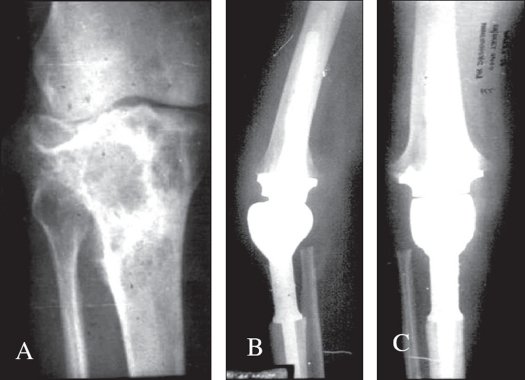
A) X-ray knee (A.P.) of a patient with GCT of proximal tibia. B), C) Ten years follow-up X-ray (Lat and AP view) after wide resection and custom mega prosthetic replacement

The average age group in our series was 30.8 years consistent with the occurrence of tumor in the third decade. The majority of our patients had soft tissue invasion and pathological fracture at presentation which may be partly due to the fact that our hospital being a tertiary referral center there was delay before the patient reported. En bloc resection of the joint and reconstruction using custom mega arthroplasty was done in all patients where skeletal reconstruction was deemed impossible [Figure 2].

One of the major technical issues of distal femoral resection is the relationship between quadriceps excision and gait which may influence implant failure and gait pattern.^33^,^34^ By following the sleeve resection of quadriceps musculature, we were able to achieve an excellent or good functional outcome in 89% of patients when compared with 84% reported by Mittermeyer *et al*.^35^ The medial gastronemius rotation flap has provided a very satisfactory method of overcoming the two major problems in managing proximal tibial tumors, namely, providing soft tissue cover for the metallic endoprosthesis and maintaining the continuity of extensor mechanism as described by Malawar and McHale *et al*.^36^ The mean extensor lag in our series was 18° (10°-35°). Extramedullary porous coated materials have shown to overcome this mechanical disadvantage.

**Graph 1:**
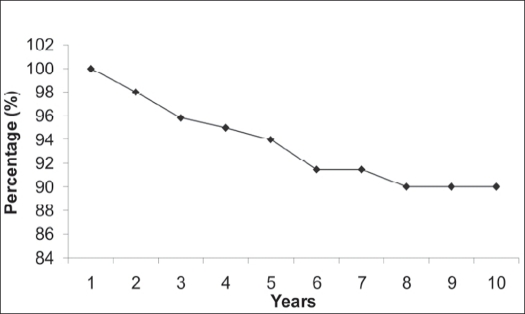
Ten-year Prosthesis survival rate as shown by Kaplan Meier estimator, X axis - Time in years, Y axis - Survival percentage

**Graph 2:**
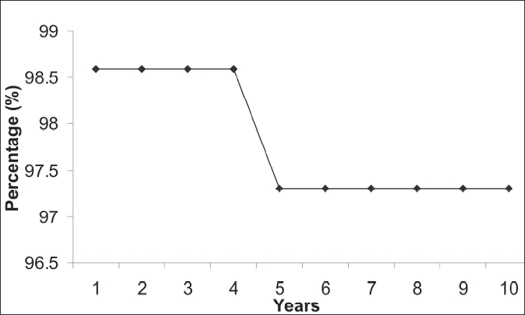
Ten-year Limb survival rate as shown by Kaplan Meier estimator, X axis - Time in years, Y axis - Survival percentage

Patients in this study belonged to a homogenous group of giant cell tumors around the knee managed by custom mega arthroplasty. The 10-year prosthetic survival in our series was 89.8% and 10-year limb survival was 97.3% [Graphs 1 and 2].

The most common complication encountered by us was periprosthetic fracture which occurred in 12 patients (8.3%) which was probably due to earlier constrained prosthesis used by us and due to the increased demands posed by return to normal activity in young patients.^31^

Infection occurred in 6.9% of our patients which is lower when compared with other series.^37^ Grimer *et al*^37^ have reported an infection rate of 33% in endoprosthetic replacement of the proximal tibia, which with improved soft tissue cover technique like the medial gastronemius flap decreased to 12%. The various factors contributing to the high rate of infection are the duration of surgery and the extensive exposure of tissues.^37^

Aseptic loosening in six (4.2%) patients was the most common late complication after cemented endoprosthetic replacement, confirming other reports.^35^,^38^ All were late failures with average duration of 5.8 years after index surgery. For four patients who had clinical symptoms of pain and instability the prosthesis was revised. The other two patients had only radiological evidence and had excellent functional outcome despite evidence of loosening. Keeping these observations we used the rotating hinge prosthesis in the majority of the patients and this design has proved to reduce the incidence of aseptic loosening.

## CONCLUSION

To conclude, achieving complete ablation of the tumor and preserving a functional extremity at the same time proves to be a daunting task due to the various anatomical factors unique to this site. Difficulties in the local control of high-grade giant cell tumors as well as high rate of local recurrence following initial surgery have led the investigators to use different surgical modalities of reconstruction. By using the technique of Limb Salvage by Custom Mega prosthesis we have been able to achieve satisfactory oncological and functional outcomes in our patients.
